# Advancing Treatment of Pituitary Adenomas through Targeted Molecular Therapies: The Acromegaly and Cushing Disease Paradigms

**DOI:** 10.3389/fsurg.2016.00045

**Published:** 2016-07-28

**Authors:** Michael A. Mooney, Elias D. Simon, Andrew S. Little

**Affiliations:** ^1^Department of Neurosurgery, Barrow Neurological Institute, St. Joseph’s Hospital and Medical Center, Phoenix, AZ, USA

**Keywords:** acromegaly, Cushing disease, endonasal, pituitary adenoma, transsphenoidal

## Abstract

The current treatment of pituitary adenomas requires a balance of conservative management, surgical resection, and in select tumor types, molecular therapy. Acromegaly treatment is an evolving field where our understanding of molecular targets and drug therapies has improved treatment options for patients with excess growth hormone levels. We highlight the use of molecular therapies in this disease process and advances in this field, which may represent a paradigm shift for the future of pituitary adenoma treatment.

## Introduction

Pituitary adenomas make up more than 90% of all pituitary tumors and are the second most commonly diagnosed non-malignant brain tumors (1). The clinical presentation of patients with pituitary adenomas is highly variable and often depends on the endocrinologic function of the tumor, the size of the tumor, or a combination of both. Given the increased use of neuroimaging studies over the past decade, a significant number of pituitary lesions are incidentally found, and the prevalence of pituitary tumors in the general population is estimated to be around 17% (2). The heterogeneity of clinical presentations combined with the relatively high prevalence of “incidentalomas” poses a diagnostic challenge to providers treating these patients, and multidisciplinary teams consisting of endocrinologists, neuro-ophthalmologists, and neurosurgeons have proven essential for delivering the highest quality of care.

Distinguishing a functional (i.e., hormone-secreting) from a non-functional adenoma is crucial for guiding subsequent treatment strategies. Although surgical resection remains the mainstay of therapy for macroadenomas causing compression of neurovascular structures, as well as for many functional microadenomas, pharmacotherapy can play a crucial role in adenoma treatment. Recent advances in genetic and molecular analysis of pituitary adenomas have provided new insights into the growth patterns and secretory functions of these tumors and have allowed for a more precise characterization of individual adenomas. These advances have led to the development of targeted molecular therapies for several subtypes of pituitary adenoma and the development of a “personalized” approach to pharmacotherapy for some patients with adenomas.

Acromegaly is one pituitary disease where recent and ongoing research has changed the standard treatment paradigm. Although pharmacotherapy has not replaced surgical resection as the mainstay of treatment, exciting advances in targeted molecular therapies have developed in recent years. We can currently implement individualized treatment options for patients with acromegaly, and the potential of this strategy is immense as our understanding of the molecular pathology of these tumors progresses. We believe that the combined surgical, targeted pharmacotherapeutic, and radiosurgical approach that is employed in acromegaly represents a paradigm that will continue to improve the treatment of not only growth hormone (GH)-secreting adenomas but also other functional and non-functional adenomas.

In this review, we highlight the current literature on the diagnosis and treatment of acromegaly with an emphasis on current targeted molecular therapies. We also review emerging treatment paradigms for Cushing disease that parallel this approach, and we discuss future directions for this exciting field.

## Growth Hormone-Secreting Adenomas

### Overview

Growth hormone-secreting pituitary adenomas manifest as the clinical syndrome acromegaly, which is a chronic disorder that results in acral overgrowth, cardiovascular disease, insulin resistance, arthritis, and sleep apnea, among other conditions (3). In children harboring a GH-secreting adenoma, excess GH production before closure of the epiphyseal plates leads to gigantism. Given the widespread effects of GH overproduction, as well as the often indolent physiologic changes in an individual patient, the diagnosis of acromegaly is often delayed. If untreated, acromegaly results in significant morbidity and increased rates of mortality for these patients (4).

The current diagnosis of acromegaly is dependent upon both the oral glucose tolerance test (OGTT) and serum levels of insulin-like growth factor-1 (IGF-1). A decline in GH production after an oral glucose load is present in normal patients, and this decrease is diminished in patients with acromegaly; detailed criteria have been established for utilizing this diagnostic tool to help identify patients with acromegaly (5). Additionally, serum IGF-1 levels are elevated in patients with acromegaly because of increased production from the liver. Circulating GH binds GH receptors on hepatocytes and activates a signaling cascade, resulting in increased in *IGF1* transcription, translation, and IGF-1 secretion (3). IGF-1 levels adequate for diagnosis are dependent on sex and age, and established values have been outlined (5–9). IGF-1 exerts effects on numerous target tissues throughout the body, and stimulation *via* this growth factor contributes to the increased morbidity and mortality encountered in patients with acromegaly (4).

Surgical resection is the mainstay of treatment for acromegaly caused by a GH-secreting adenoma. However, not all patients are candidates for surgery, and not all adenomas are amenable to complete resection. Since surgical treatment is not always an option, a large role for both pharmacotherapy and stereotactic radiosurgery has developed in this population of patients. An improved molecular understanding of pituitary adenomas has advanced pharmacologic options for acromegaly patients, and we hypothesize that this is the start of a paradigm shift in the treatment of acromegaly. In this article, we review the literature on surgical success rates and targeted molecular therapies in acromegaly. Radiosurgery success rates and expert opinions on the implementation of this strategy have been reviewed elsewhere (10–12).

### Surgical Treatment

Surgical success rates in the literature vary widely depending on tumor size, the degree of invasion, surgeon experience, adjuvant therapies, and definition of success (i.e., laboratory values defined as curative). When examining the success of surgery alone, the largest series as of 2016 examined 688 patients with acromegaly treated at a single center (13). Criteria required to define a cure included normalization of basal GH to <2.5 ng/L, suppression of GH to <1 ng/L during the OGTT, and IGF-I normal for age and sex, which are the current standard definitions for biochemical remission. The overall remission rate for all tumors treated *via* the transsphenoidal approach was 57.3% at the 3-month follow-up in this study. Of note, success varied widely based on tumor size and invasion characteristics, with 75.3% of microadenomas surgically in remission versus 41.5% of macroadenomas with parasellar or sphenoidal extension. In this series, only two patients with surgical remission developed recurrent acromegaly within a mean follow-up of 10 or more years.

Numerous smaller series in the literature largely support these values, with surgical remission rates ranging to 60% (14–20). Reported recurrence rates in the literature to date vary widely due to the different criteria for biochemical remission and varying years of follow-up; recurrence rates ranging from 0.4 to 19% (7, 13, 17, 21–23) are reported, with one 2012 meta-analysis citing a mean 6% recurrence rate within 10 years (20).

### Targeted Molecular Therapies

For the subset of acromegaly patients without biochemical remission after surgery, or for those patients who are unable or unwilling to undergo surgery, pharmacotherapy takes on an essential role. Pharmacotherapy for acromegaly was first used in the 1970s, and our understanding of GH-secreting adenomas has significantly advanced since that time (24). With a better understanding of the molecular biology of GH-secreting cells, the introduction of more targeted therapies has been possible, and we can now better tailor pharmacotherapy regimens for individual patients with acromegaly.

The population of cells within the anterior pituitary gland that secrete GH were identified in the early twentieth century in association with acromegaly and became known as somatotroph cells (24). Like other cell types of the anterior pituitary gland, somatotroph cells typically remain under tight physiologic control through positive and negative feedback from the hypothalamus. Somatotroph cells express two classes of receptors that mediate negative feedback – dopamine receptors (DRs) and somatostatin receptors. Both pathways have been successfully targeted pharmacologically and with a resultant decrease in GH secretion in patients with acromegaly. A third pathway, the GH receptor pathway, has also been successfully targeted for acromegaly pharmacotherapy. All three pathways are reviewed here.

Dopamine receptors are encoded by five separate genes (*DRD1–DRD5*). However, *DRD2* and *DRD4* are the two genes predominantly expressed in the normal pituitary gland (25). *DRD2* is strongly expressed in both somatotrophs and lactotrophs, and binding of dopamine (or dopamine agonist medications) to DRD2 triggers an inhibitory signaling cascade to decrease prolactin secretion. DRs were first targeted in the 1970s with the dopamine agonist bromocriptine; however, the dopamine agonist cabergoline has since proven to be more effective due to its increased DR2 selectivity and longer half life (26, 27). Interestingly, DR2 expression levels in somatotrophs are correlated with dopamine agonist response rates both *in vitro* and *in vivo*, and analysis of prolactin and DR2 expression patterns within GH-secreting adenomas has been proposed as a guide for pharmacotherapy strategies in acromegaly patients (28–31). Furthermore, dopamine agonists are recommended for adenomas that secrete both GH and prolactin if pharmacotherapy is needed after surgery because both expression pathways are targeted by these agents (32).

Somatostatin receptors are also encoded by five separate genes (*SSTR1–SSTR5*), and the *SSTR2* and *SSTR5* subtypes make up 90–95% of receptor expression in GH-secreting adenomas (33). *SSTR* expression is found within normal pituitary cells including corticotrophs and lactotrophs, and binding of somatostatin to SSTRs triggers a G-protein-mediated signal cascade that inhibits secretory function in these cells. The two standard somatostatin analogs in use today are octreotide and lanreotide, which activate this signaling pathway to inhibit hormone production in functional adenomas. There is significant heterogeneity in clinical responsiveness to these agents, and recent research suggests this may be due to heterogeneous *SSTR* subtype expression between patients (34, 35). More recently, the somatostatin analog pasireotide was developed, which has increased binding affinity for SSTR2 and SSTR5 compared to octreotide and lanreotide. Pasireotide has shown superior efficacy for biochemical control in some studies of patients with acromegaly (36). This drug class is one example of how an improved molecular understanding of somatotrophs may provide more efficacious treatment options for patients with acromegaly; however, further studies are required before receptor expression profiles can be used to guide clinical practice.

The class of GH-receptor antagonists is the third and final example of successful targeted molecular therapy in acromegaly. GH receptors are found primarily in the liver and cartilage where activation triggers the JAK–STAT (Janus kinase/signal transducers and activators of transcription) pathway and ultimately leads to upregulation in cell proliferation and antiapoptotic proteins, including IGF-1 (3). Pegvisomant is currently the only GH-receptor antagonist approved by the U.S. Food and Drug Administration that is available for treatment of acromegaly, and it is a pegylated analog of human GH, which directly competes for receptor binding with plasma GH (37). Binding of pegvisomant prevents dimerization of the GH-receptor and thereby blocks the signaling cascade, resulting in decreased IGF-1 production. Of note, this mechanism is significantly different from that of the dopamine and somatostatin analogs, because it blocks the downstream effects of a GH-secreting adenoma, independent of tumor receptor expression patterns (3, 38). Use of pegvisomant is typically reserved for patients in whom treatment with somatostatin analogs fails or in patients with diabetes mellitus (32, 39).

### Current and Future Clinical Practice

Complete surgical resection of a GH-secreting adenoma remains the first-line treatment option for acromegaly today. Surgical cure rates are high, with low morbidity and mortality when surgery is performed at a center with an experienced neurosurgical team as the first-line treatment for eligible patients. In patients with persistent or recurrent disease after surgery, or those unable to or unwilling to undergo surgery, pharmacotherapy and stereotactic radiosurgery remain excellent treatment options. Several pathways of pharmacotherapy for acromegaly have been evaluated, including use as adjuvant therapy (following surgery), as neoadjuvant therapy (before surgery), and as primary therapy (in place of surgery). The efficacy and implementation of stereotactic radiosurgery for functional pituitary adenomas have been extensively reviewed elsewhere (10–12).

Numerous studies have evaluated the efficacy of pharmacotherapy for persistent or recurrent disease after surgical resection, and somatostatin analogs are considered first-line therapy for these patients (40). It is estimated that approximately 30–60% of patients with persistent disease after surgical resection achieve biochemical remission with the addition of a somatostatin analog (41–44). An additional percentage of patients achieve biochemical remission with dopamine agonists, pegvisomant, or combination therapy with these agents. Radiological follow-up in these patients must be interpreted with caution. Tumor shrinkage is often observed with postoperative somatostatin analog treatment, but, this does not reliably correlate with biochemical remission (42, 45). Notably, some studies have noted correlations in somatostatin and dopamine expression patterns in the adenoma with treatment response, which may allow for a more individualized approach to pharmacotherapy strategies in these patients in the future (2, 29, 30, 34, 35, 46–49). GH and IGF-1 must be closely monitored in patients with known residual tumor undergoing adjuvant treatment, and treatment strategies for recurrent disease must be made on a case-by-case basis.

Neoadjuvant therapy with somatostatin analogs has been attempted in patients with large GH-secreting adenomas with some success. Preoperative treatment with somatostatin analogs was investigated in multiple studies of macroadenomas secreting GH, and this regimen was consistently shown to decrease tumor volume and GH secretion levels in patients prior to surgery (50). Additionally, short-term biochemical remission rates (3–4 months postoperatively) were consistently improved with neoadjuvant therapy. However, this effect was not clearly demonstrated for long-term remission rates, and further studies on this subject remain to be performed. Although preoperative somatostatin analogs may decrease tumor volume, they do not convert unresectable, invasive tumors into resectable lesions. We believe this may limit the success of this strategy going forward, and preoperative somatostatin analogs should be given only to a small subset of carefully selected patients.

Since their introduction, the success of monotherapy with somatostatin analogs for some patients has been the most impressive contribution of pharmacotherapy for acromegaly. Recent studies have demonstrated good biochemical control with such treatment (36, 51–53). When interpreting biochemical control data, it is important to consider whether the patient population was preselected for somatostatin responsiveness and what other treatments the patients have received. The published remission rates for somatostatin analogs have declined as more experience is gained with the drugs because of the increased recognition of these two factors. Although most studies to date have focused on octreotide or lanreotide monotherapy, we hypothesize that future studies investigating new-generation somatostatin analogs, such as pasireotide, could demonstrate superior results. Preliminary studies with pasireotide show significantly higher rates of biochemical control compared with octreotide (36, 54).

At our institution, we attempt complete resection as the first-line treatment. In patients with residual disease not amenable to further resection (and with elevated GH and IGF-1 levels postoperatively), adjuvant somatostatin analog therapy is initiated, and patients are monitored for their biochemical response. Concurrent adjuvant radiosurgery with somatostatin analog treatment is provided on a case-by-case basis considering the location and volume of the residual tumor.

**Figure 1:**
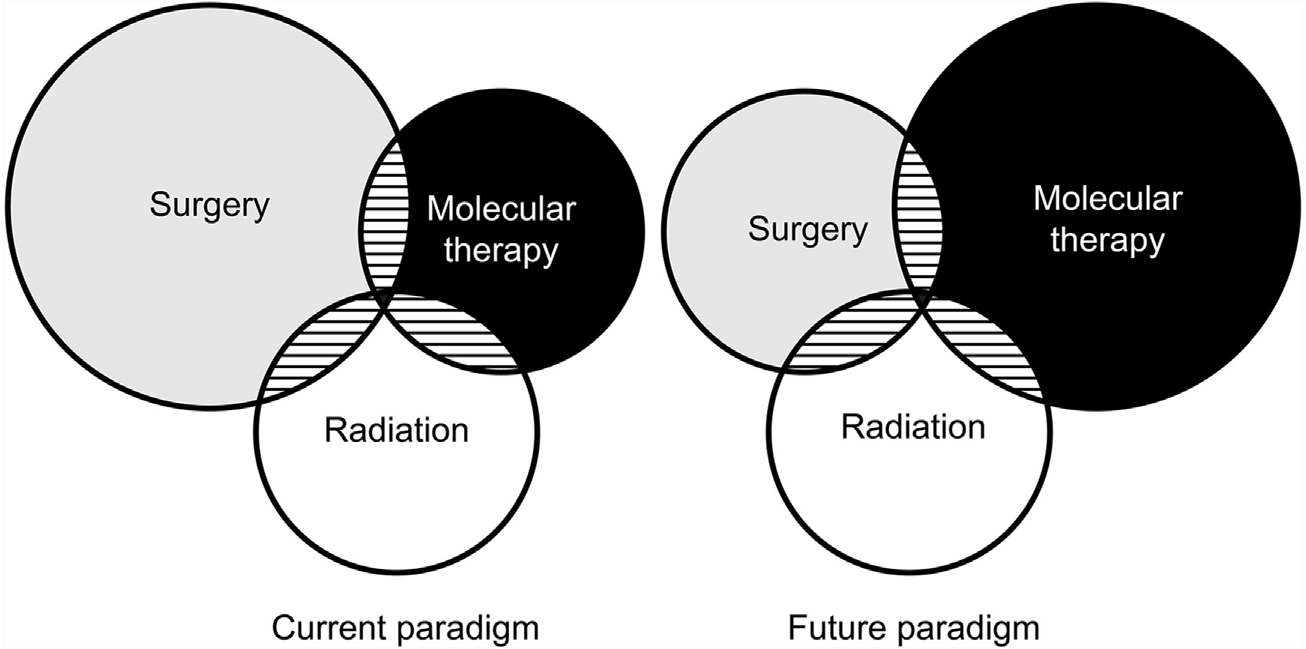
**Venn diagram illustrating the relative impact of antitumor treatments in acromegaly as practiced today and what it might look like in the future**. Used with permission from Barrow Neurological Institute, Phoenix, Arizona.

As our molecular understanding of somatotrophs advances and drugs are developed to target new sites, the role for pharmacotherapy in acromegaly will continue to expand. Although surgical resection remains the mainstay of treatment today, the future likely holds a shift in our treatment paradigm to one that emphasizes pharmacotherapy (Figure 1). Personalized approaches to pharmacotherapy for acromegaly may emerge based on molecular expression profiles of individual patient tumors; however, future research on this subject is required before it can be used to guide treatment for patients with acromegaly.

## Other Functional Adenomas

### Cushing Disease

Adrenocorticotropic hormone (ACTH)-secreting pituitary adenomas manifest as the clinical syndrome of Cushing disease. Excessive secretion of ACTH leads to hypercortisolemia, and these patients present with widespread clinical symptoms including central obesity, facial plethora, amenorrhea, and skin changes, among many others. Surgical resection of the ACTH-secreting adenoma is currently the mainstay of treatment for patients with Cushing disease. However, as with acromegaly, the role for pharmacotherapy in treated Cushing disease is growing. As our molecular understanding of this disease progresses, drug development continues to produce new treatment options for patients with persistent or recurrent disease after surgery, as well as for those patients unable or unwilling to undergo surgery.

Today, the rate of postoperative biochemical remission following surgical resection of microadenomas is approximately 75% while remission of macroadenomas is about 43% (55–57). For the remainder of patients and for those who do not undergo surgery, the addition of pharmacotherapy plays a crucial role in treatment. Pharmacotherapy of Cushing disease targets three main pathways: central secretory action at the level of the pituitary, steroidogenesis, and end-target action at the glucocorticoid receptor (58). As with acromegaly, increasing knowledge of corticotroph receptor expression has guided medical treatment options for this disease.

Corticotrophs express high levels of *SSTR5* and *DRD2*, similar to the expression seen in somatotrophs (59). Pharmacotherapy targets these receptors using somatostatin ligands and dopamine agonists, respectively, to decrease ACTH production by corticotroph adenomas. Pasireotide, in particular, has demonstrated efficacy in patients with Cushing disease due to its relatively high binding affinity for SSTR5 (60). Phase II and III clinical trials utilizing pasireotide in patients who have not undergone surgery have demonstrated a significant reduction in urinary free cortisol levels, as well as improvement in symptoms of hypercortisolemia (61). A large, randomized, double-blind, multicenter, phase III study is currently underway to evaluate pasireotide as monotherapy for this group of patients. Cabergoline is also used in the medical management of Cushing disease by targeting corticotroph secretory function. Several trials have demonstrated its efficacy both *in vitro* and *in vivo* (62–64). In non-responders or partial responders to a single agent, combination therapy with pasireotide and cabergoline, was shown to be effective in decreasing urinary free cortisol levels (65). Other somatostatin analogs and dopamine agonists (octreotide, lanreotide, and bromocriptine) are not as effective in Cushing disease as they are in acromegaly, and these agents are not routinely used in clinical practice today.

Ketoconazole and metyrapone are the most widely used steroidogenesis inhibitors prescribed today for refractory Cushing syndrome; however, no prospective studies have evaluated these agents in Cushing disease, and their use is currently off-label (66). Investigations into alternative steroidogenesis targets are ongoing and may hold future promise (67). Mifepristone is the only current glucocorticoid receptor antagonist available for use in Cushing disease, and it is Food and Drug Administration approved for treatment of hyperglycemia in Cushing syndrome (61). It has demonstrated efficacy in long-term symptom resolution in a multicenter trial (68); however, its use is contraindicated in pregnant women, and it may be associated with adenoma enlargement (69). Serial magnetic resonance imaging is warranted to monitor for such enlargement in patients with Cushing disease treated with mifepristone.

Although numerous pharmacologic targets exist in Cushing disease, medical management has yet to reach the efficacy and safety of surgery; transsphenoidal resection remains the treatment of choice for eligible patients with an ACTH-secreting adenoma. Similar to acromegaly, an improved understanding of the molecular basis of corticotroph cells and end-target receptors will continue to spur drug development and improve medical treatment options for this challenging disease.

## Conclusion and Future Directions

Pituitary adenomas are relatively common tumors, and transsphenoidal resection is a safe and effective treatment option for many of these lesions. Surgical resection by an experienced pituitary surgeon remains the mainstay of therapy for both acromegaly and Cushing disease. However, a significant percentage of patients have persistent or recurrent disease after surgery or are not surgical candidates. An improved understanding of the molecular biology of these diseases has evolved since the mid-1970s, and targeted molecular therapies that limit the growth, secretory function, and end-organ effects of these tumors continue to be developed. The greatest success has come with the class of somatostatin analogs, and new knowledge regarding receptor subtype expression in pituitary adenomas has helped guide treatment strategies. Further research into this domain may allow for more individualized treatment strategies for patients harboring tumors with expression patterns that can be characterized. Although some research has supported this approach to date, further studies are required before this paradigm can be applied outside of academic pituitary practices. Characterization of tumor expression patterns is a challenging task, but we believe that targeted pharmacotherapy could approach, and eventually surpass, the efficacy of surgical resection for the treatment of these lesions.

## Author Contributions

All authors have made substantial, direct, and intellectual contributions to the work and approved it for publication.

## Conflict of Interest Statement

Dr. AL has an ownership interest in Kogent.

## Abbreviations

ACTH, adrenocorticotropic hormone; DR, dopamine receptor; GH, growth hormone; IGF, insulin-like growth factor; OGTT, oral glucose tolerance test.
